# Bladder, Bowel, and Sexual Dysfunction in Parkinson's Disease

**DOI:** 10.4061/2011/924605

**Published:** 2011-09-12

**Authors:** Ryuji Sakakibara, Masahiko Kishi, Emina Ogawa, Fuyuki Tateno, Tomoyuki Uchiyama, Tatsuya Yamamoto, Tomonori Yamanishi

**Affiliations:** ^1^Neurology Division, Department of Internal Medicine, Sakura Medical Center, Toho University, 564-1 Shimoshizu, Sakura 285-8741, Japan; ^2^Department of Neurology, Chiba University, Chiba 263-8522, Japan; ^3^Department of Urology, Dokkyo Medical University, Tochigi 321-0293, Japan

## Abstract

Bladder dysfunction (urinary urgency/frequency), bowel dysfunction (constipation), and sexual dysfunction (erectile dysfunction) (also called “pelvic organ” dysfunctions) are common nonmotor disorders in Parkinson's disease (PD). In contrast to motor disorders, pelvic organ autonomic dysfunctions are often nonresponsive to levodopa treatment. The brain pathology causing the bladder dysfunction (appearance of overactivity) involves an altered dopamine-basal ganglia circuit, which normally suppresses the micturition reflex. By contrast, peripheral myenteric pathology causing slowed colonic transit (loss of rectal contractions) and central pathology causing weak strain and paradoxical anal sphincter contraction on defecation (PSD, also called as anismus) are responsible for the bowel dysfunction. In addition, hypothalamic dysfunction is mostly responsible for the sexual dysfunction (decrease in libido and erection) in PD, via altered dopamine-oxytocin pathways, which normally promote libido and erection. The pathophysiology of the pelvic organ dysfunction in PD differs from that in multiple system atrophy; therefore, it might aid in differential diagnosis. Anticholinergic agents are used to treat bladder dysfunction in PD, although these drugs should be used with caution particularly in elderly patients who have cognitive decline. Dietary fibers, laxatives, and “prokinetic” drugs such as serotonergic agonists are used to treat bowel dysfunction in PD. Phosphodiesterase inhibitors are used to treat sexual dysfunction in PD. These treatments might be beneficial in maximizing the patients' quality of life.

## 1. Introduction

Parkinson's disease (PD) is a common movement disorder associated with the degeneration of dopaminergic neurons in the substantia nigra. In addition to the movement disorder, patients with PD often show nonmotor disorders. The nonmotor problems of PD include neuropsychiatric disorders, sleep disorders, sensory symptoms, and autonomic disorders [1]. Bladder, bowel, and sexual dysfunction (also called “pelvic organ” dysfunctions) is one of the most common autonomic disorders [2, 3]. Studies have shown that the pelvic organ dysfunctions have great significance in relation to quality-of-life measures, early institutionalization, and health economics [4, 5]. It is particularly important to note that, unlike motor disorder, pelvic organ dysfunctions are often nonresponsive to levodopa, suggesting that they occur through a complex pathomechanism [6]. This is because pathology of PD is not confined to the degeneration of dopaminergic neurons in the substantia nigra, and involves other locations in the brain and other neurotransmitter systems than the dopaminergic system. For this reason, add-on therapy is required to maximize patients' quality of life. This article reviews pelvic organ dysfunctions in PD, with particular reference to neural control of the bladder [2], bowel [2], and genital organs, symptoms, objective assessment, and treatment.

## 2. Bladder Dysfunction in PD

### 2.1. Neural Control of Micturition: Normal Micturition and Detrusor Overactivity

**Figure 1:**
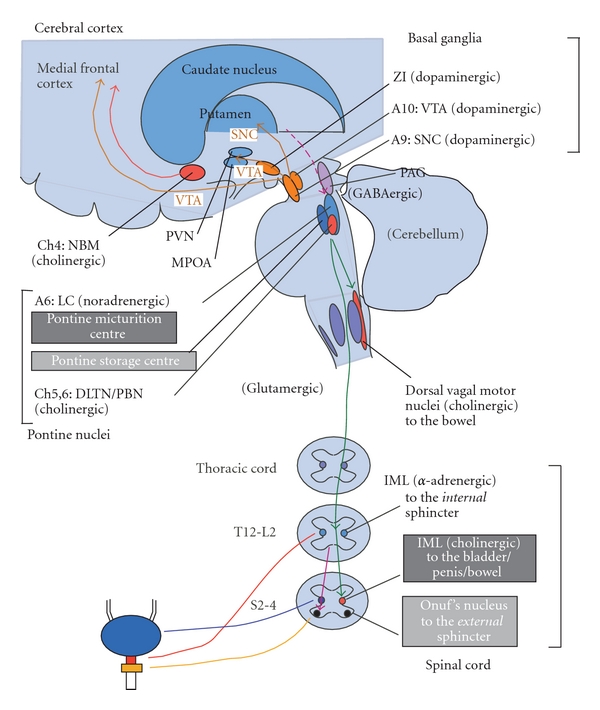
Neural circuitry relevant to micturition. PAG, periaqueductal gray; LC, locus ceruleus; NBM, nucleus basalis Meynert; PVN, paraventricular nucleus; MPOA, medial preoptic area; A, adrenergic/noradrenergic; ZI, zona incerta; VTA, ventral tegmental area; SNC, substantia nigra pars compacta; DLTN, dorsolateral tegmental nucleus; PBN, parabrachial nucleus; IML, intermediolateral cell column; GABA, *γ*-aminobutyric acid; T, thoracic; L, lumbar; S, sacral. See text.

The lower urinary tract (LUT) consists of two major components, the bladder and urethra. The bladder has abundant muscarinic M2, 3 receptors and adrenergic beta 3 receptors, and is innervated by cholinergic (parasympathetic) and noradrenergic (sympathetic) fibers for contraction and relaxation, respectively [7]. The urethra has abundant adrenergic alpha 1A/D receptors and nicotinic receptors, and is innervated by noradrenergic (sympathetic; contraction) and cholinergic (somatic; contraction) fibers (Figure 1). The LUT performs two opposite functions, storage and emptying of urine, both of which require an intact neuraxis that involves almost all parts of the nervous system [8]. This is in contrast to postural hypotension, which arises due to lesions below the medullary circulation center in humans [9].

Normal urinary storage is dependent on the sacral autonomic reflex [7, 10]. The storage reflex is thought to be tonically facilitated by the brain, particularly the pontine storage center [11, 12]. The pontine storage center lies just ventrolateral to the pontine micturition center (PMC). In addition to the pontine storage center, the storage function is facilitated by the hypothalamus, cerebellum, basal ganglia, and frontal cortex. These areas have been shown to be activated during urinary storage by functional neuroimaging in humans [13]. Normal micturition is dependent on the spino-bulbo-spinal autonomic reflex [7], which particularly involves the midbrain periaqueductal gray matter (PAG) [14–17] and the PMC [7, 11]. The PAG is thought to be central in regulating micturition and has a range of inputs from the higher structures. The PMC is located in or adjacent to the locus coeruleus [18–20]. The PMC is thought to project spinal descending fibers containing glutamate as a facilitatory neurotransmitter, which activates the sacral bladder preganglionic nucleus [21]. PMC also projects fibers containing *γ*-amino-butyric acid (GABA) and glycine as inhibitory neurotransmitters, which suppresses the sacral urethral motor nucleus (the Onuf's nucleus) [22]. The voiding function seems to be initiated and facilitated by the higher brain structures, for example, the hypothalamus and prefrontal cortex, which seem to overlap in the storage-facilitating area [13, 23]. Bladder (detrusor) overactivity (DO) is the major cause of urinary urgency/frequency and incontinence [24]. In lesions above the brainstem, the micturition reflex arc is intact, where DO is considered an exaggerated micturition reflex [24–26]. The exaggeration of the micturition reflex might be brought about by more than simply the decreased inhibition of the brain, and might be further facilitated by glutamatergic and D2 dopaminergic mechanisms [27].

### 2.2. Basal Ganglia Circuit and Dopamine

**Figure 2:**
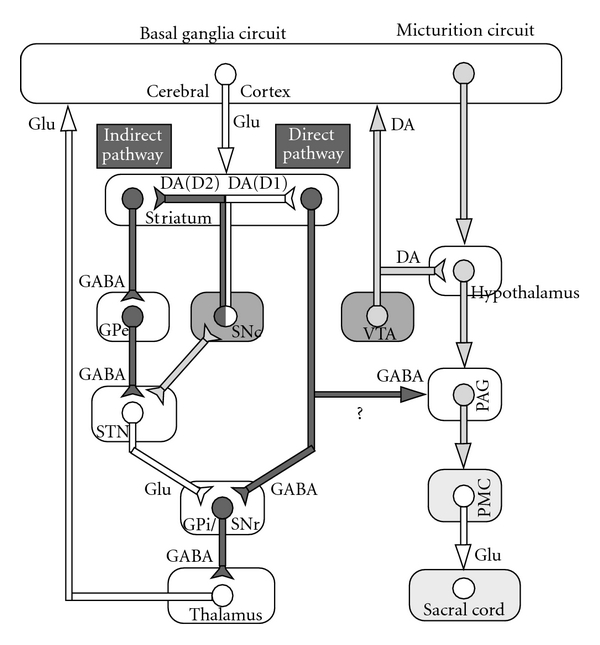
Possible relationship between basal ganglia circuit (left side) and micturition circuit (right side; modified from Sakakibara et al. [39]). DA, dopamine; GABA, gamma-aminobutyric acid; SNc, substantia nigra pars compacta; GPi, globus pallidus internus; SNr, substantia nigra pars reticulate; STN, subthalamic nucleus; GPe, globus pallidus externus; VTA, ventral tegmental area; PMC, pontine micturition centre; Glu, glutamate; black line, inhibitory neurons; white line, excitatory neurons; hatched line, neurons of undetermined property. The micturition reflex (right-side pathway) is under the influences of dopamine (DA; both inhibitory in D1 and facilitatory in D2) and gamma-aminobutyric acid (GABA; inhibitory). The substantia nigra pars compacta (SNc) neuronal firing and the released striatal dopamine seem to activate the dopamine D1-GABAergic *direct pathway*, which not only inhibits the basal ganglia output nuclei (e.g., the globus pallidus internus (GPi), substantia nigra pars reticulata (SNr)), but also may inhibit the micturition reflex via GABAergic collateral to the micturition circuit. High-frequency stimulation (leading to inhibition) in the subthalamic nucleus (STN) also results in bladder inhibition. See text.

The net effect of the basal ganglia on micturition is thought to be inhibitory (Figure 2) [7, 28–30]. Functional neuroimaging during bladder filling results in activation in the globus pallidus of normal volunteers [31] and in the putamen in patients with PD [32]. In contrast, dopamine transporter imaging was lower in PD patients with urinary dysfunction than in those without it [33, 34]. Electrical stimulation of the substantia nigra pars compacta (SNc) inhibited the micturition reflex [35, 36], and striatal dopamine levels *in situ* significantly increased in the urinary storage phase in experimental animals [37]. The micturition reflex is under the influences of dopamine (both inhibitory in D1 and facilitatory in D2) and GABA (inhibitory) [7, 28]. Both the SNc neuronal firing and the released striatal dopamine seem to activate the dopamine D1-GABAergic *direct pathway* (Figure 2), which not only inhibits the basal ganglia output nuclei, but also may inhibit the micturition reflex via GABAergic collateral to the micturition circuit [37–40]. In patients with PD, disruption of this pathway may lead to DO and resultant urinary urgency/frequency. In addition to the nigrostriatal fibers, the ventral tegmental area (VTA)-mesolimbic dopaminergic fibers are thought to be involved in the control of micturition [36, 41, 42] (Figure 1).

### 2.3. Bladder Dysfunction in PD

#### 2.3.1. Lower Urinary Tract Symptom

The reported prevalence of LUT symptoms (LUTS) in patients with PD ranges from 38% to 71% [43–48]. However, it has been difficult to determine to what extent PD contributes to LUTS. Men older than 60 years of age may have bladder outlet obstruction due to prostate hyperplasia. Women may have stress urinary incontinence. “Idiopathic DO” [10] may occur in men and women older than 65 years due in part to latent brain ischemia [49]. Some of the studies were published before the diagnosis of multiple system atrophy (MSA) [50] was recognized. In recent studies of PD patients who were diagnosed according to modern criteria [5, 51–53], the prevalence of LUTS was found to be 27–63.9% using validated questionnaires [51–53], or 53% in men and 63% in women using a nonvalidated questionnaire that includes a urinary incontinence category [5], with all of these values being significantly higher than the incidence rates in healthy controls. The majority of patients had onset of bladder dysfunction after appearance of motor disorder. Correlations have been shown between bladder dysfunction in patients with PD and neurological disability [51], and bladder dysfunction and stage of disease [5], both suggesting a relationship between dopaminergic degeneration and LUTS. However, Campos-Sousa and colleagues did not find such a correlation [53].

#### 2.3.2. Storage Symptoms

LUTS are divided majorly into two; storage symptoms and voiding symptoms. Storage symptoms are the most common of the LUTS symptom types in PD. Storage symptoms include nocturia (nighttime urinary frequency), which is the most prevalent symptom reported by patients with PD (>60%) [5, 51–53]. Patients also complain of urinary urgency (33–54%) and daytime frequency (16–36%). Urinary incontinence was present in 26% of male and 28% of female patients with PD [5].

#### 2.3.3. Voiding Symptoms

Although less common than storage symptoms, voiding symptoms also occur in PD patients. In the study by Sakakibara and colleagues, PD patients had significantly higher rates of retardation in initiating urination (44% of men only), prolongation/poor stream (70% of men only), and straining (28% of women only) compared with the control group [5]. Araki and colleagues noted a correlation between voiding symptoms and stage of disease [54]. However, despite the voiding symptoms, PD patients have low postvoid residuals [5].

#### 2.3.4. Videourodynamics, Pressure-Flow Analysis, and Sphincter Electromyography

**Bladder (Detrusor) Overactivity**

**Figure 3:**
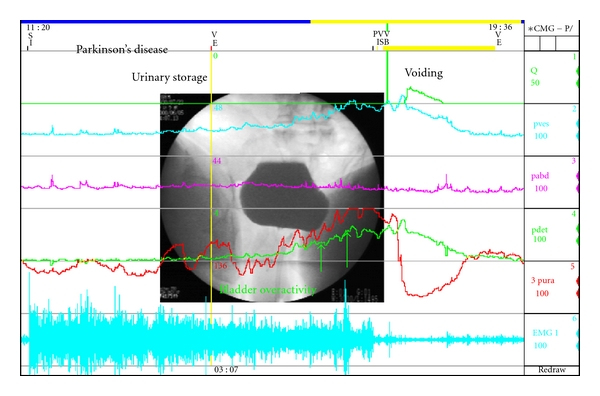
Detrusor (bladder) overactivity by urodynamic measurement.

The storage-phase urodynamic abnormalities in PD include reduced bladder capacity together with detrusor overactivity (DO) in 45–93% [43, 44, 54–58] of patients, and uninhibited external sphincter relaxation in 33% [53] of patients (Figure 3). Therefore, DO can be the major contributing factor to overactive bladder in PD. There is also a correlation between DO and stage of disease [55].

**Mild, Weak Detrusor, and Sphincter Obstruction**

Pressure-flow analysis [10, 59, 60] of the voiding phase in PD has shown weak detrusor activity during voiding (40% of men; 66% of women) [56]. There is a correlation between a weak detrusor and the stage of the disease [55]. A subset of PD patients had DO during storage but weak detrusor activity in voiding. This combination has recently been estimated to occur in 18% of patients with PD [61]. Some older studies described detrusor-external sphincter dyssynergia or pseudodyssynergia in PD, and these findings were attributed to PD by analogy with bradykinesia of the limbs [62]. However, in our patients with PD, detrusor-external sphincter dyssynergia was rare [56]. In contrast, a pressure-flow analysis in PD revealed that half of the patients with PD showed mild urethral obstruction [56]. Patients with PD are reported to have high resting urethral pressure, probably as a result of medication—that is, levodopa and its metabolites, such as norepinephrine. Irrespective of voiding symptoms in PD, the average volume of postvoid residuals in PD was as small as 18 mL [56].

**Differential Diagnosis of Parkinsonism by Bladder Dysfunction**

In the differential diagnosis of PD and parkinsonian-type MSA, large postvoid residuals, open bladder neck, and neurogenic change in sphincter motor unit potentials are all common in MSA [56, 63] whereas they are rarely seen in clinically typical PD. However, recent evidence suggests that PD with dementia, or dementia with Lewy bodies [64], may have large postvoid residuals and neurogenic change in the sphincter motor unit potentials [65], thereby mimicking MSA.

### 2.4. Treatment of Bladder Dysfunction in PD

#### 2.4.1. Dopaminergic Drugs

It is possible that levodopa and other antiparkinson medication may affect bladder function in PD. Aranda and Cramer [66] studied the effects of 3–8 mg apomorphine injection on the storage function in 2 *de novo* PD patients, and found that the bladder capacity increased. They gave oral levodopa to one of the patients, and the bladder capacity increased. We compared the frequency of bladder dysfunction in *de novo* PD and PD with levodopa. In that study, LUTS was less frequent than in the treated group [59]. In another study, after 3 months of treatment with levodopa, the storage urodynamic parameters were slightly improved in *de novo* PD [67].

In contrast, in treated patients, studies concerning the effect of dopaminergic drugs on micturition have produced conflicting results. Regarding overactive bladder, some reports have shown a storage-facilitating effect of dopaminergic drugs [5]. In contrast, Kuno and colleagues showed that a change in medication from bromocriptine (D2 selective agonist) to pergolide (D1 < 2 agonist) brought lessening of nocturia [68], and Yamamoto described improvement of DO by pergolide [69]. Benson and colleagues [70] gave 2000 mg of levodopa in 2 longstanding PD patients, and bladder capacity increased in both patients. After discontinuation of levodopa, the bladder capacity further increased in one of the patients, but decreased in the other. Other reports have shown a voiding-facilitating effect of dopaminergic drugs [71]. Fitzmaurice and colleagues [72] have described that, in advanced PD with the on-off phenomenon, DO worsened with levodopa in some patients and lessened in others. Winge and colleagues [73] found that the effect on micturition of treatment with dopaminergic drugs in PD was unpredictable. Recent studies have shown that in early PD [74] and advanced PD with the on-off phenomenon [6], a single dose of levodopa exacerbates DO in the filling phase. We still do not know the exact reasons for the discrepancy.

There are several factors underlying the complex bladder behavior in treated PD patients [75]. Postsynaptic dopamine D1 (excitatory) and D2 (inhibitory) receptors have a millimolar affinity to dopamine whereas dendritic D2 (inhibitory) autoreceptors have a picomolar affinity to dopamine [76]. Therefore, levodopa may first stimulate dendritic D2 autoreceptors, which might suppress the dopaminergic cells and facilitate the micturition reflex. In cases of PD under long-term treatment with levodopa, dopamine receptors are downregulated and potential hypersensitivity might occur [77]. The A11 dopaminergic cell group lies in the dorsal-posterior hypothalamus, which is affected in marmosets with MPTP-induced parkinsonism [78]. This cell group descends as the sole source of spinal dopamine [79], which might also involve in generating bladder overactivity [80]. Peripheral dopamine D1 and D2 receptors also exist in the bladder [81], although their exact role has not been delineated.

#### 2.4.2. Cholinergic Drugs

Anticholinergics [82] are generally used as a first-line treatment for overactive bladder. However, it is important to balance the therapeutic benefits of these drugs with their potential adverse effects. When the dose of drug increases, postvoid residuals may appear [75]. Dry mouth and constipation are common [83]. Cognitive adverse events by anticholinergics are a concern particularly in the elderly. For example, trihexyphenidyl (for PD) and oxybutynin (for overactive bladder) have been shown to have central side effects [84, 85]. Factors contributing to the central effects of drugs may include blood-brain barrier (BBB) penetration [86]. Among the factors of BBB penetration, diffusion is facilitated by lipophilicity [87]. Particularly in elderly patients who have hallucinations or cognitive decline (PD with dementia/dementia with Lewy bodies) [64, 65], anticholinergics should be used with extreme caution.

#### 2.4.3. Other Treatments

When a first-line treatment fails or is contraindicated, a second-line treatment might be considered. The main action of central 5-hydroxytryptamine- (5-HT, or serotonin-) ergic neurons on the LUT is facilitation of urine storage [88]. In PD, neuronal cell loss in the raphe nucleus has been documented [89]. Therefore, serotonergic drugs, such as duloxetine and milnacipran [90] can be a choice to treat overactive bladder in PD. Nocturnal polyuria should be distinguished from overactive bladder. In patients with PD, the imbalance between diurnal and nocturnal production of urine can be observed in the course of the disease [91]. Treatment with desmopressin proved to be effective in reducing nocturia in PD [92], although this medication needs caution of water intoxication. The subthalamic nucleus (STN) is regarded as the key area in the *indirect* pathway, which is dominant in the parkinsonian state [93]. Deep brain stimulation (DBS) in the STN inhibits many cells within the STN, probably due to depolarization block and release of GABA from activation of inhibitory afferent terminals [94]. In the STN, neuronal firings related to the micturition cycle have been observed in cats [39]. DBS in the STN proved to have an inhibitory effects on the micturition reflex in animals [39, 40] and in patients with PD [95–97]. DBS in the STN also increased bladder capacity and facilitated bladder afferent pathways in the brain of PD patients [98, 99].

## 3. Bowel Dysfunction in PD

### 3.1. Neural Control of Defecation: Enteric Nervous System and Dopamine

**Figure 4:**
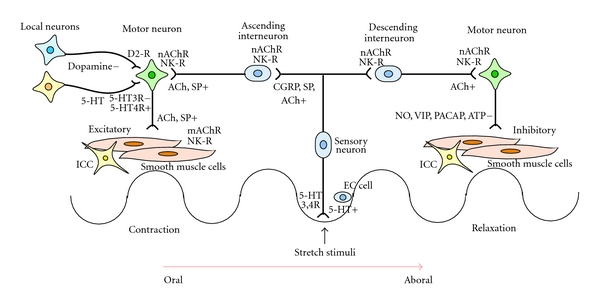
Enteric neural circuitry relevant to peristaltic reflex. Following mucosal stimulation, 5-HT is released from enterochromaffin cells to intrinsic primary sensory neurons (with 5-HT3 and 5-HT4 receptors) and extrinsic vagal and spinal sensory neurons (with 5-HT3 receptors). Sensory neurons release calcitonin gene-regulated peptide (CGRP), substance (SP), and acetylcholine (ACh) to interneurons. Interneurons release ACh and SP orally to excitatory motorneurons while ACh is released aborally to inhibitory motorneurons. Excitatory motorneurons release ACh and SP to smooth muscle cells while inhibitory motorneurons release nitric oxide (NO), vasoactive intestinal peptide (VIP), pituitary adenylate cyclase-activating polypeptide (PACAP), and adenosine triphosphate (ATP) to smooth muscle cells. 5-HT also acts as an excitatory modulator on motor neurons (with 5-HT3 and 5-HT4 receptors) whereas dopamine seems to be an inhibitory modulator on motor neurons (with D2 receptor). Interstitial cells of Cajal (ICC) interact with smooth muscle cells for generating rhythmicity (with Ach, VIP, and NO receptors).

The enteric nervous system (ENS) plays the most important role in regulating the peristaltic reflex of the lower gastrointestinal (GI) tract (LGIT) [100]. Slow phasic pressure waves are the most common manometric phenomenon [101], and are measured in the colon and rectum (spontaneous phasic rectal contraction) in humans [102–105]. The origin of the slow wave rhythmicity in LGIT has been identified in the myenteric (Auerbach's) and submucous (Meisner's) plexuses, where interstitial cells of Cajal (ICC) exist [106]. The peristaltic reflex consists of two components: ascending contraction oral to, and descending relaxation caudal to, the site of stimulus (Figure 4). The reflex can be evoked by surface stroking or by circumferential stretch [100], in which 5-HT stimulates the sensory nerve terminals [107]. The oral excitatory component is mediated by cholinergic fibers whereas the aboral inhibitory component is mediated by nonadrenergic, noncholinergic fibers. Thus, local neuronal circuits and ICCs, together with appropriate external stimuli, might bring about the peristaltic reflex. Other types of pressure changes in the colon include giant motor complexes [100], which is perhaps analogous to the migrating motor complex of small intestine [100, 101]. After a meal, the motility index increases for 20 to 30 min and remains elevated for up to 3 hours. A combination of slow waves and giant motor complexes is thought to promote bowel transport, which is measured by colonic transit time (CTT) in humans [107, 108].

The strength of cholinergic transmission in the ENS is thought to be regulated by opposing receptors; serotonin 5-HT4 receptor-mediating excitation [109, 110] and dopamine D2 receptor-mediating inhibition [111, 112]. Endogenous 5-HT may facilitate intestinal motility [109], as colorectal motility is greater than normal in 5-HT transporter knockout mice with elevated extracellular 5-HT levels. Reports using dopamine transporter knockout mice have indicated that endogenous dopamine may inhibit intestinal motility [113, 114]. However, a number of studies have also demonstrated increased motility in the colon (scarce in dopamine receptors) in response to dopamine [115, 116], presumably mediated by other receptor populations such as adrenergic or serotonergic receptors, or by central mechanisms [116]. It is uncertain whether MPTP-induced parkinsonian animals might have enteric dopaminergic pathology as seen in PD [117–121]. Nevertheless, MPTP/salsolinol-induced parkinsonian animals showed decreased GI motility [122] and decreased c-Kit expression in the ICC [123]. More recently, Dorolet and colleagues found myenteric plexus alpha-synuclein aggregate pathology, neuron loss, and slowing of gastrointestinal motility in rotenone-induced parkinsonian animals [124].

### 3.2. Extraenteric Nervous System and Dopamine

**Figure 5:**
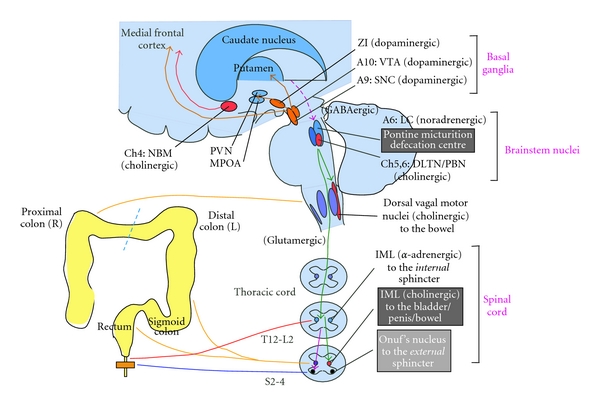
Neural circuitry relevant to defecation. Although less significantly than the lower urinary tract (LUT) does, it is thought that function of the lower gastrointestinal tract depends on the brain and the spinal cord. Whereas small intestine and ascending colon are innervated by the vagus nerves originating in the medulla, descending colon, sigmoid colon, and rectum primarily share sacral innervation of the LUT (Figure 1). Both the sacral cord and the vagus nuclei receive projecting fibers from Barrington's nucleus (the pontine micturition/defecation center). Bowel function seems to be modulated by the higher brain structures; including the frontal lobe, the hypothalamus, and the basal ganglia; the main action of the latter on the bowel seems to be inhibitory. NBM: nucleus basalis Meynert, Ch: cholinergic, PVN: paraventricular nucleus, MPOA: medial preoptic area, ZI: zona incerta, A: adrenergic/noradrenergic, VTA: ventral tegmental area, SNC: substantia nigra pars compacta, LC: locus ceruleus, DLTN: dorsolateral tegmental nucleus, PBN: parabrachial nucleus, PAG: periaqueductal gray, IML: IML cell column, GABA: *γ*-aminobutyric acid, T: thoracic, L: lumbar, S: sacral.

Whereas small intestine and ascending colon are innervated by the vagus nerves originating in the medulla, extraenteric innervation of descending colon, sigmoid colon, and rectum primarily shares that of the LUT (Figure 5) [7, 102]. LUT and LGIT perform the similar function of storage and emptying. However, there are also profound differences with regard to physiology (dysfunctional transport, rare ureter versus common bowel; smooth muscle contraction, only on emptying bladder contraction versus persistent spontaneous phasic rectal contraction; abdominal strain, minimum versus large, resp.) [102]. In addition, while the LUT requires intact neuraxis for storage and emptying [7], it has not been entirely clear to what extent LGIT needs extra-ENS.

Acute transection of the pelvic plexus shows slowed transit and abolishes the defecation reflex [125]. Six months later, the transit and defecation reflex are recovered [125]. In contrast, chronic replacement of esophagus [126] or bladder [127] by a colonic segment preserves colonic motility. Pathological studies in PD have shown a degenerative lesion in the spinal parasympathetic PGN [128], although the degree is much less than that in MSA. No Lewy bodies were found in the Onuf's nucleus innervating the anal sphincter [128]. Both the sacral cord and the vagus nuclei receive projecting fibers from Barrington's nucleus (identical to the PMC) in the pons. The spinal descending pathway for defecation is located in the lateral columns in humans [129, 130]. In the acute stage of spinal cord injury [131] or multiple sclerosis [132], CTT is significantly prolonged. In the chronic phase, prolonged CTT of the proximal colon returns to normal whereas that of the distal colon persists [133, 134]. Abdominal strain and cough are accompanied by sphincter contraction, which is called the guarding reflex [135]. However, when the sphincter contraction is large enough, defecation becomes unsuccessful as commonly seen in spinal cord injury (paradoxical sphincter contraction on defecation (PSCD) or anismus) [136]. In the brainstem, lesion in the vagus nuclei causes intestinal pseudo-obstruction [137, 138]. In PD, neuronal cell loss [139] and the appearance of Lewy bodies [128] in the vagus nuclei have been documented. Barrington's nucleus is thought to be critical to eliciting migrating motor complex [140, 141]. In PD, involvement of the Barrington's nucleus has also been documented [139].

The basal ganglia modulate the bowel motility [142, 143], with the main action apparently being inhibitory [142, 143]. However, under stress conditions, facilitatory responses were also observed [144, 145]. Although the connection is not fully clarified, bowel function seems to be modulated by the higher brain structures [146]. Most areas activated in functional neuroimaging by bowel distention [147] strikingly overlap the area activated by bladder distention [148].

### 3.3. Bowel Dysfunction in PD

#### 3.3.1. Lower Gastrointestinal Tract Symptoms

In PD there is dysfunction along the entire length of the GI tract. Therefore, while we focus on the colon and rectum, we refer to stomach and small intestine when necessary. The reported prevalence of LGIT symptoms in PD is mostly more than half [149–151]. However, it has been difficult to determine the extent to which PD is contributing to the LGIT symptoms. This difficulty occurs because not only PD but also idiopathic constipation may occur in the elderly due in part to dietary habit [151], exercise [152], or age-related ENS degeneration [153]. Controlled studies [5, 149, 154, 155] could overcome these problems, in which the incidence rate of decreased stool frequency (<3 times a week) in PD patients ranges from 20% to 81%, that of difficulty in stool expulsion in 57–67%, and that of diarrhea in 21%. All of these values are significantly higher than in the normal population (range, decreased stool frequency, 0–33%; difficulty in stool expulsion, 26–28%; diarrhea, 10%) [5, 149, 154, 155]. Fecal incontinence has been reported to be 10–24% in PD [5, 149]. Therefore, constipation is the most prominent LGIT symptoms in patients with PD. Indeed, PD is a risk factor for elderly nursing home residents to have constipation [156]. Of particular importance is that bowel dysfunction affects the quality of life in patients with PD [5].

There is no significant difference in the use of dopaminergic or anticholinergic drugs and bowel dysfunction [5, 149]. Difficulty in expulsion, and diarrhea are more common in the higher grade of Hoehn and Yahr staging [5, 149, 150], suggesting a relationship between dopaminergic degeneration and LGIT symptoms. However, there are also studies in which no such relationship was found [157]. Constipation in PD occurs commonly with a low coefficient of variation in electrocardiographic R to R intervals [158]. The findings indicate that parasympathetic dysfunction might underlie these abnormalities. A recent epidemiological study revealed an association between the frequency of bowel movements and the future risk of developing PD [159]. From a clinical perspective, it is of particular importance that patients with PD see gastroenterologists or physicians first because of their bowel dysfunction before a diagnosis of PD is made.

A more severe and often acute presentation of bowel dysfunction is intestinal pseudo-obstruction [160], also called paralytic ileus. Yokoyama and Hasegawa [161] have recently reported the frequency to be 7.1% among 112 patients with PD. Radiographical features of intestinal pseudo-obstruction are dilatation of colon and small intestine [160, 162–164]. True obstruction due to volvulus in PD also occur [161, 163, 164]. Intestinal pseudo-obstruction also occurs insidiously [165, 166]. In such patients, histology specimens may reveal Lewy body disorder.

#### 3.3.2. Lower Gastrointestinal Tract Function Tests

LGIT function primarily consists of (1) colonic transport of the bowel content to the anorectum [107, 108], (2) transient anorectum reservoir, and (3) defecation from the anorectum with the aid of strain [102]. In PD, constipation results primarily from decreased transport and/or disturbed anorectal evacuation. Fecal incontinence may result from disturbed anorectal reservoir, or overflow secondary to constipation.

#### 3.3.3. Transit Time Study

Previous reports have shown that total CTT is increased beyond the normal threshold in 80% of PD patients [167], which translates into an increased average CTT ranging from 44 hours to 130 hours in PD [167, 168], and in 89 hours in de novo PD patients [167], all of which are significantly longer than those of controls (range, 20–39 hours) [160, 167, 168]. Prolonged CTT has also been documented in PD patients without subjective constipation [169]. Slow colonic transit is the major cause of decreased stool frequency. The slow colonic transit is likely to reflect a decrease in slow waves and spike activities of the colon, which may reflect the ENS pathology, and to a lesser extent, the CNS pathology in PD. Among right, left, and rectosigmoid segments of the CTT, the rectosigmoid CTT is significantly prolonged in PD patients [160, 167, 169]. Several explanations for this finding can be hypothesized. It is possible that the ENS innervated by the sacral cord is more severely affected than that innervated by the DMV in PD [160]. Oro-caecal transit time in PD is also prolonged [170].

Pathological studies have demonstrated that PD affects the ENS [117–121]; showing decrease in dopaminergic myenteric neurons and the appearance of Lewy bodies along the proximal-distal axis, for example, they were most frequent in the lower esophagus, but scarce in the rectum. Presumably, degeneration of not only the inhibitory (dopaminergic) fibers, but also of facilitatory (cholinergic and serotonergic) fibers might contribute to the slow colonic transit in PD.

#### 3.3.4. Rectoanal Videomanometry and Sphincter Electromyography

**Resting State**

Anal function at the resting state is measured by anal manometry and analysis of the external sphincter motor unit potentials. At the resting state, the anal pressure of PD patients is low or normal [160, 171]. The resting anal pressure may reflect sympathetic innervation in the internal anal sphincter, since lesions or anaesthetic blocks at T12-L3 (where the sympathetic PGN is located) substantially lessen the anal pressure [172]. Similarly, PD patients have low [173] or normal [160, 171] anal pressure increase on squeezing. However, neurogenic changes in motor unit potentials of the external sphincter muscles occur in only 0–15% of PD patients [174, 175]. This negative finding may correspond to the result of pathological studies indicating that the sacral Onuf's nucleus is spared in the majority of PD patients [128]. Nevertheless, the latent anal sphincter dysfunction may explain the fecal incontinence that occurs in most advanced cases. The rectoanal inhibitory reflex comprises a slow anal pressure decrease following a rapid distention of the intra-rectal balloon [176]. This reflex might be appropriate for evacuation. In PD, the threshold of the rectoanal inhibitory reflex is reduced [168, 171, 177] or normal. Although this reflex is thought to be mediated by the intrinsic ENS [178], the exact reflex arc is not entirely clear.

#### 3.3.5. Filling Phase

Rectoanal videomanometry measures functions of the anorectum reservoir and evacuation. During slow rectal filling, PD patients have a slightly but not significantly larger rectal volume at first sensation and a maximum desire to defecate compared with control subjects [160, 171]. The PD patients had the same rectal compliance as control subjects using the slow-liquid-filling method [102, 160], which is in accordance with the studies using the balloon-inflation method. However, the amplitude of the spontaneous phasic rectal contraction in the PD patients is significantly less than that in control subjects [102, 160]. The decreased spontaneous phasic rectal contraction may share the same aetiology with the decrease in CTT.

In normal subjects, anal pressure not only varies during the storage phase, but also shows a close relation with spontaneous phasic rectal contraction, for example, when the rectal pressure increases, the anal pressure tends to decrease [102]. The concurrent sphincter relaxation with the spontaneous phasic rectal contraction resembles the rectoanal inhibitory reflex [176]. The concurrent sphincter relaxation might be appropriate for the following evacuation phase [102]. However, in the PD patients, both rectal and anal pressures tend to increase together [160]. This phenomenon during filling resembles the paradoxical sphincter contraction on defecation, as described below.

#### 3.3.6. Defecation Phase

In addition to slow transit constipation, anorectal (outlet type) constipation is a common feature in PD. Indeed, most PD patients could not defecate completely and had postdefecation residuals, the volume of which were significantly larger than those in a control group [160]. During defecation, it has remained a subject of controversy whether true rectal contraction occurs, since abdominal strain is large enough to mask the rectal contraction if present. Only a few studies have measured the differential rectal pressure component [179] and not found rectal contraction on defecation. In recent studies, healthy control subjects had a moderate rectal pressure increase on defecation, for example, the healthy subjects utilized the final wave of spontaneous phasic rectal contractions for defecation [102]. However, rectal contraction on defecation in PD patients is smaller than that in controls [160].

The abdominal straining in PD patients is less than that in control subjects [160]. Straining plays a physiological role in both coughing and defecation, which is achieved by cocontraction of the glottis, diaphragm, and abdominal wall [180]. Straining is associated with activation in brainstem nuclei such as the Kolliker-Fuse nucleus and medullary respiratory neurons [180]. However, PD patients show a less pronounced increase in abdominal pressure on coughing [160, 181] and Valsalva maneuver [160, 182] before starting rectal filling, and in the defecation phase [160, 182], than do control subjects. The mechanism of the impaired straining in PD may include rigidity and reduced contractility of the axial muscles, and a failure of coordinated glottis closure [181]. However, neuronal degeneration in the CNS relevant to straining is yet to be clarified in PD.

During defecation, the anal pressure increase on defecation in patients is significantly larger than that in control subjects, with an increase in the sphincter electromyography (EMG) activity. This finding in PD has been described as paradoxical sphincter contraction on defecation (PSCD), or anismus [118, 160, 167, 171, 182, 183]. During fictive defecation (straining), a lack of anal inhibition has also been reported in 65% of PD patients. Although PSCD can also be seen in patients with idiopathic constipation [184], the frequent occurrence of PSCD in PD suggests that PSCD is a disease-related condition. The frequency of PSCD is almost the same in early and late PD [185], indicating that PSCD is an early defecatory abnormality. Mathers and colleagues [182] consider PSCD a focal dystonia. PSCD also occurs in spinal cord-injured patients [136], suggesting that dysfunction in the suprasacral descending pathway to the external sphincter is a contributing factor. Apomorphine is shown to lessen PSCD [182, 183]. This effect was not antagonized by domperidone, which did not penetrate the BBB, suggesting that the CNS pathology may produce PSCD. Both weak abdominal strain and PSCD seem to be the major causes of difficulty in stool expulsion in PD patients.

### 3.4. Treatment of Bowel Dysfunction in PD

#### 3.4.1. Dietary Fibers

Although it is not certain whether exercise may facilitate bowel habit in PD, in the healthy population, moderate exercise is reported to shorten mouth-to-anus transit time [186] and improve overall wellbeing [152]. Water content is an important determinant to make stools normal (70% water) or hard (40–60% water) [184]. PD patients are reported to have reduced water intake [187]. Diet and laxatives are the first-line treatment for constipation [188]. Dietary fibers such as psyllium produced an improvement in stool consistency and an increase in stool frequency in healthy population [189] and PD [169, 190]. Polyethylene glycol 3350 [191], or bulking and highly hydrophilic agent polycarbophil [192], improve constipation in PD.

#### 3.4.2. Cholinergic Drugs

A prior report has shown that pyridostigmine bromide, an acetylcholinesterase inhibitor, is effective in the amelioration of constipation in PD [193].

#### 3.4.3. Dopaminergic Drugs

**Levodopa and Other Dopaminergic Agonists**

Dopamine is used as a peripheral vasoactive drug in intensive care units, in which dopamine is shown to reduce gastric migrating motor complex [194]. Dopamine also decreases gastric motility in normal volunteers [195] whereas it increases motility of the duodenum and sigmoid colon. Similarly, dopamine administration increases colonic motor activity in irritable bowel syndrome [196]. Unlike dopamine, levodopa penetrates the BBB [197]. However, it is possible that levodopa acts on the ENS and affects bowel function in PD, since levodopa can also be metabolized in the periphery. A modern formula utilizes levodopa in combination with peripheral dopa-decarboxylase inhibitor. This regimen could possibly reduce GI side effects [198]. However, no reports are available to see whether levodopa might change gut function in untreated PD patients. As for somatic sphincter function, levodopa improves voluntary anal squeezing in fluctuating PD patients, which parallels an improvement in gait difficulty from “off” to “on” stage [177]. Apomorphine, a dopaminergic agonist, has also been shown to lessen PSCD [182, 183]. This effect is not antagonized by domperidone, suggesting that apomorphine might act on the CNS dopaminergic pathways.

#### 3.4.4. Dopaminergic Blockers

Dopaminergic blockers (domperidone, etc.) are widely used as GI prokinetics by means of antagonizing dopamine's inhibitory effects on the GI motility, particularly D2 receptor blockade [199]. The pharmacological profiles of the compounds differ in terms of their molecular structure, affinity at D2 receptors, and ability to interact with other receptor systems (5-HT3 and 5-HT4 receptors for metoclopramide; 5-HT4 receptors for levosulpiride). Since domperidone does not cross the BBB, it can be used as GI prokinetics for constipation in PD [200], although the effect of domperidone on constipation is minimal. In contrast, dopaminergic blockers that could penetrate the BBB (metoclopramide, levosulpiride, etc.) may potentially worsen extrapyramidal motor disorder in PD [199]. Since levodopa is absorbed from the small intestine [201], bowel dysfunction in PD may interfere with levodopa absorption, worsen the motor disorder, or even lead to malignant syndrome [202, 203]. Gastric emptying of an isotope-labeled solid meal becomes significantly faster during domperidone therapy in PD [200]. In addition, domperidone pretreatment causes a mean 12% increase in peak plasma levodopa concentrations that occurs a mean of 10 min earlier than when levodopa is given alone [202]. Peak plasma levodopa concentrations are reported to be greater on levodopa-domperidone than on levodopa-carbidopa [204].

#### 3.4.5. Serotonergic Drugs

Cisapride, a selective 5-HT 4 receptor agonist, has significantly shortened CTT in PD [205], although after 1 year, only a small effect could be demonstrated [206]. Of particular importance is that cisapride add-on therapy improved the “on-off” phenomenon in advanced PD [203]. However, some reports indicated cisapride's D2 dopaminergic receptor blocking property [207]. Cisapride also blocks K^+^ channels and leads to cardiotoxicity. Mosapride is a novel selective 5-HT 4 receptor agonist that lacks a D2 receptor or K^+^ channel blocking property [208]. Mosapride is shown to ameliorate delayed gastric emptying [209] as well as constipation in PD [210], by shortening of total CTT (particularly the caudal segment), and by augmenting the amplitude in rectal contraction during defecation [210]. Improvement of parkinsonism is more significant with pergolide-mosapride than with pergolide-domperidone [211]. Tegaserod, a selective 5-HT 4 agonist, is also effective in ameliorating constipation in PD [212].

#### 3.4.6. Other Drugs

Although prior reports have indicated the effectiveness of motilides (erythromycin, etc.) [213], neurotrophin-3 [214] and colchicine [215] on constipation in PD, their use remains limited. Type A botulinum toxin injection into the puborectalis muscle [216, 217] and biofeedback [218] ameliorates anismus in PD.

## 4. Sexual Dysfunction in PD

### 4.1. Neural Control of Erection: Normal Erection in Men

Sexual dysfunction is not uncommon in PD [5, 219–223]. Studies have shown that sexual dysfunction has great significance in relation to quality-of-life measures. However, the detailed mechanism of sexual dysfunction in PD has not been well known. 

**Figure 6:**
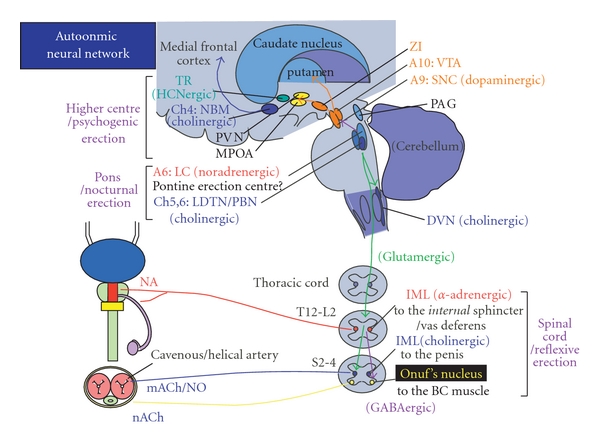
Neural circuitry relevant to erection. PAG, periaqueductal gray; LC, locus coeruleus; NBM, nucleus basalis Meynert; PVN, paraventricular nucleus; MPOA, medial preoptic area; A, adrenergic/noradrenergic; ZI, zona incerta; VTA, ventral tegmental area; SNC, substantia nigra pars compacta; DLTN, dorsolateral tegmental nucleus; PBN, parabrachial nucleus; IML, intermediolateral nucleus; GABA, *γ*-aminobutyric acid; T, thoracic; L, lumbar; S, sacral; NA, noradrenaline; Ach, acetylcholine; NO, nitric oxide. See text.

The genital organ primarily shares lumbosacral innervation with the lower urinary tract. Erection is a vascular event [224]; occurring secondarily after dilatation of the cavernous helical artery and compression of the cavernous vein to the tunica albuginea [224]. Helical artery dilatation is brought about by activation of cholinergic and nitrergic nerves; this activation facilitates nitric oxide secretion from the vascular endothelium. Ejaculation is brought about by contraction of the vas deferens and the bladder neck, in order to prevent retrograde ejaculation, by activation of adrenergic nerves (Figure 6). Sexual intercourse in healthy men can be divided into 3 phases [225]: (a) desire (libido), (b) excitement and erection, and (c) orgasm, seminal emission from the vas deferens, and ejaculation from the penis. Erection can be further classified into 3 types by the relevant stimulation: (1) psychogenic erection (by audiovisual stimulation), (2) reflexive erection (by somatosensory stimulation), and (3) nocturnal penile tumescence (NPT; associated with rapid eye movement (REM-) sleep). “Morning erection” is considered the last NPT in the nighttime.

### 4.2. Hypothalamic Neurons and Dopamine in Men

Among the 3 types of erection, reflexive erection requires an intact sacral cord, particularly the intermediolateral (IML) cell columns. Pathology studies have shown that involvement of the IML nucleus is common in MSA, whereas it is uncommon in PD. Therefore, reflexive erection can be affected in patients with MSA. In patients with a supra-sacral spinal cord lesion, reflexive erection might be preserved, whereas psychogenic erection is severely disturbed because of a lesion in the spinal pathways to the sacral cord. Libido and erection are thought to be regulated by the hypothalamus; particularly the medial preoptic area (MPOA) and the paraventricular nucleus (PVN) (Figure 4) [226, 227]. Electrical or chemical stimulation in the MPOA/PVN evoked erection and mating behaviors in experimental animals, both of which were abolished by destruction of these areas. Somatosensory inputs from the genitalia ascend in the anterior spinal cord, and project to the MPOA/PVN via the thalamic nuclei. Erotic visual inputs from the retina are thought to reach the MPOA via the mamillary body. Recent neuroimaging studies have shown that penile stimulation or watching pornography activated these areas in humans [228]. NPT [229] seems to be regulated by the hypothalamic lateral preoptic area [230].

The raphe nucleus and the locus ceruleus are candidate areas participating in the regulation of NPT. Oxytocinergic neurons in the hypothalamic PVN are thought to facilitate erection by projecting directly to the sacral cord, and by projecting to the midbrain periaqueductal gray and the Barrington's nucleus (identical to the PMC). Serum oxytocin concentration increases during masturbation in healthy men. 

In experimental animals, dopamine is known to facilitate erection and mating behaviors. The MPOA/PVN receives projections from the nigral dopaminergic neurons [231]. A microdialysis study showed that the dopamine concentration in the MPOA was increased by sexual stimulation. It is reported that dopamine D1/D2 receptors in the hypothalamus participate in erection whereas only D2 receptors participate in ejaculation. Pathology studies have shown that the hypothalamus is affected in PD [231]. Recently, polymorphism in the dopamine D4 receptor gene is shown to contribute to individual differences in human sexual behavior [232]. Prolactinergic neurons are thought to be inhibitory in sexual function. Serum prolactin levels increase after orgasm in healthy men. Prolactin-producing pituitary tumors often cause gynecomastia and erectile dysfunction in male patients. Hyperprolactinemia occurs after the use of sulpiride, metoclopramide, and chlorpromazine (all dopamine receptor antagonists). Therefore, dopaminergic neurons seem to facilitate oxytocinergic neurons whereas they inhibit prolactinergic neurons. Some *de novo* PD patients have hyper prolactinemia [233], which may contribute to erectile dysfunction in those patients.

### 4.3. Female Sexual Function and Dopamine

As compared with male genitalia, studies of female genital organs are limited. Vulva [234], clitoris [235] and vagina [236] primarily shares lumbosacral innervation with the lower urinary tract. Sexual arousal in women is a vasocongestive and neuromuscular event through these organs, paralleling genital lubrication, controlled by facilitatory parasympathetic (by vasoactive intestinal peptide, nitric oxide, and to a lesser extent acetylcholine, via the pelvic nerves from S2–4 intermediolateral (IML) cell column) and inhibitory sympathetic (by noradrenaline, via the hypogastric nerves from T12-L3 IML cell column,) inputs. Some information is thought to travel the vagal nerves. Activity of these spinal nuclei is controlled by sensory afferents from the genitalia and descending projections from the brain.

Like men, libido in women is thought to be regulated by the hypothalamus. The neural circuit for lordosis in animals involves a supraspinal loop, which is controlled by an estrogen- and progesterone-dependent signal, presumably at the ventromedial hypothalamus (VMH), medial preoptic area (MPOA), and paraventricular nucleus (PVN) in the hypothalamus. Lordosis is facilitated by lutenizing hormone-releasing factor (LHRH), alpha-melanocyte stimulating hormone (alpha-MSH), and methionine-enkephalin whereas suppressed by corticotrophin-releasing factor and beta-endorphin [237, 238]. Recent neuroimaging studies have shown that vaginal self-stimulation with/without orgasm activated these areas in humans [239]. Oxytocinergic neurons in the VMH and the MPOA are thought to facilitate vagino-clitorial sexual arousal and lordosis by projecting directly to the sacral cord [238, 240]. Role of dopamine in female sexual function remains not completely clear [241]. It is reported that lordosis was increased in female animals by microinjection of apomorphine (D1, 2 agonist) and quinpirole (D2 agonist) in the MPOA whereas it decreased by SKF 38393 (D1 agonist) [242]. This was counteracted by chemical inhibition of dopaminergic neurons in the ventral tegmental area (VTA) [243].

#### 4.3.1. Male Sexual Symptoms

The reported prevalence of sexual symptoms in men with PD ranges from 37% to 65% [234–239]. Only few previous studies have looked at sexual symptoms in PD and control subjects. Jacobs et al. [244] studied 121 men with PD (mean age 45 years) and 126 age- and sex-matched community male controls. Patients were more dissatisfied with their present sexual functioning and relationship whereas no differences were found for the frequency of sexual intercourse itself. Erection and ejaculation were not inquired. Sakakibara et al. [5] analyzed sexual function of 46 men with PD (age 35–70 years old) and 258 healthy male control subjects (age 30–70 years old) [5]. As compared with the control group, the frequency of dysfunction in PD patients was significantly higher for decrease of libido (84%), decrease of sexual intercourse (55%), decrease of orgasm (87%), and decrease of erection (79%) and ejaculation (79%). Therefore, sexual dysfunction is significant in PD. The majority of patients had onset of the sexual dysfunction after the appearance of the motor disorder. This is in contrast to patients with MSA, who often have sexual dysfunction before the onset of motor disorder.

Comparing the results between four age subgroups (subjects in their 30s, 40s, 50s, and 60s) in the control group, the frequencies of sexual intercourse and of orgasm were significantly lower in older individuals [5]. In the PD group, only the frequency of orgasm was lower in older men (*P* < 0.05). Comparing the results between both sexes in the control group, decrease of libido and orgasm were more common in women (*P* < 0.01). In the PD group, there was no significant difference in sexual function items. Bronner et al. [245] reported that use of medications (selective serotonin reuptake inhibitors used for comorbid depression), and advanced PD stage contributed to the development of ED.

#### 4.3.2. Rigiscan

In healthy men, sexual intercourse is thought to be carried out by integrating affective, motor, sensory, autonomic, and other factors. In male patients with PD, depression, motor disorder, and pain inevitably lead to sexual dysfunction. In contrast, it has been difficult to determine to what extent autonomic factors contribute to the sexual dysfunction in PD. However, erectile dysfunction often precedes motor disorder in MSA, and abnormal NPT is not uncommon in PD. These findings strongly suggest that the disorder does in fact contribute to sexual dysfunction in PD. Rigiscan is an objective measure for erectile dysfunction, which allows both tumescence and rigidity measurement, and is suitable for assessing NPT.

Only few data have been available concerning the relationship between NPT and dopamine. However, in experimental animals, administration of levodopa elicited erection and yawning together. Animals with experimental parkinsonism showed fewer REM stages during sleep than control animals did.

#### 4.3.3. Female Sexual Dysfunction

As compared with men, few studies are available concerning female sexual dysfunction in PD. Further, only few previous studies have looked at female sexual symptoms in PD and control subjects. Sakakibara et al. [5] analyzed sexual function of 38 women with PD (age 35–70 years old) and 98 healthy control women (age 30–70 years old) [5]. As compared with the control group, the frequency of dysfunction in women with PD was significantly higher for decrease of libido (83%) and decrease of sexual intercourse (88%) while decrease of orgasm was not different between women with PD and control. The majority of patients had onset of the sexual dysfunction after the appearance of the motor disorder. Welsh et al. [246] studied 27 women with PD (mean age 67 years) and community healthy controls, and in both group 50% were sexually active. As compared with control, women with PD had more common decrease of libido, vaginal tightness, involuntary urination, and dissatisfaction in sexual intercourse. There was no difference in terms of sexual arousal, sexual intercourse, and orgasm. Without control, in young PD patients (36–56 years) women had more common sexual problems (decrease of libido, 70%, decrease of sexual intercourse, 80%) than men (40%, 33.4%, resp.) [247, 248]. Other domains, such as loss of lubrication and pain, are not clearly known.

### 4.4. Treatment of Sexual Dysfunction in PD

#### 4.4.1. Male Sexual Dysfunction

**Dopaminergic Drugs**

It is possible that levodopa and other antiparkinson medication may affect sexual function in PD. However, it is not entirely clear to what extent levodopa ameliorates sexual dysfunction in PD. In contrast, subcutaneous apomorphine injection is used to ameliorate fluctuating symptoms in PD. It has also been used to treat erectile dysfunction in the general population [247, 248] and in patients with PD [249], although the dose is different (general population, initial 2 mg and up to 3 mg [247, 248], PD, 4 mg [250]). Apomorphine is thought to stimulate dopamine D2 receptors, and activate oxytocinergic neurons in the PVN. Nausea is a common side effect of this drug. Cabergoline [251] and pergolide [252] are also reported to improve sexual dysfunction in PD. In contrast, pathological hypersexuality may occur together with [253] or without delirium [254], which is attributed to the dopamine dysregulation syndrome in this disorder. DBS in the STN has produced either improved sexual wellbeing [255] or transient mania with hypersexuality [256] in patients with PD.

**Phosphodiesterase-5 Inhibitors**

When dopaminergic drugs did not help, phosphodiesterase-5 inhibitors, for example, sildenafil, vardenafil, and so forth, become the first line treatment in PD [257, 258]. These drugs inhibit nitric oxide degradation and facilitate smooth muscle relaxation in the cavernous tissue. When treating PD patients with postural hypotension, these drugs should be prescribed with extreme caution [258].

**Other Drugs**

When phosphodiesterase-5 inhibitors did not help, recent trials of melanocortin, for example, melanotan-II, bremelanotide, and so forth, showed that these drugs might become a choice for treating erectile dysfunction. A group of pro-opio-melanocortin (POMC) gene products include adrenocorticotropic hormone (ACTH), *α*-melanocyte stimulating hormone (*α*-MSH), *β*-MSH, and *γ*-MSH. It is known that the arcuate nucleus of the hypothalamus projects POMC-containing neurons to the lateral hypothalamus, dorsal medial nucleus, MPOA, and PVN [22]. *α*-MSH as secreted in the MPOA and PVN participates in the central control of sexual function [21]. Bremelanotide is a melanocortin receptor agonist, and is reported to be effective for treating erectile dysfunction as compared with placebo [259].

#### 4.4.2. Female Sexual Dysfunction

There is no established regimen to treat sexual dysfunction in women with PD. PDE5 inhibitors, such as sildenafil, tadalafil, and vardenafil, has become the first choice in the treatment of erectile dysfunction in men. Whereas sildenafil facilitated clitorial engorgement in women with sexual dysfunction [260], clinical efficacy of this drug in women with PD awaits further clarification [261]. Bremelanotide, a melanocortin receptor agonist, is applied to female sexual dysfunction, and is reported to be effective [262].

## 5. Conclusions

This article reviewed the current concepts of bladder, bowel, and sexual dysfunction (pelvic organ dysfunctions) in PD. Central nervous system pathology is clearly associated with bladder (urinary urgency/frequency) and sexual dysfunction (decrease in libido, erection, and overall dissatisfaction) in PD. In contrast, both central (weak strain and anismus) and peripheral myenteric pathology (slow colonic transit and loss of rectal contraction) are associated with bowel dysfunction. Anticholinergic agents are generally used to treat bladder dysfunction while phosphodiesterase inhibitors are used to treat sexual dysfunction. Dietary fibers, laxatives, and serotonergic agents are used to treat bowel dysfunction. These treatments are beneficial in maximizing patients' quality of life.
